# Ascorbyl Palmitate Hydrogel for Local, Intestinal Delivery of Macromolecules

**DOI:** 10.3390/pharmaceutics10040188

**Published:** 2018-10-15

**Authors:** Jinyi Zhai, Julia Mantaj, Driton Vllasaliu

**Affiliations:** School of Cancer and Pharmaceutical Sciences, Faculty of Life Sciences & Medicine, King’s College London, London SE1 9NH, UK; jinyi.zhai@kcl.ac.uk (J.Z.); julia.mantaj@kcl.ac.uk (J.M.)

**Keywords:** ascorbyl palmitate, biologics, biologics delivery, hydrogels, inflammatory bowel disease, intestinal delivery

## Abstract

Biologics have changed the management of inflammatory bowel disease (IBD), but there are concerns with unexpected systemic toxicity and loss of therapeutic response following administration by injection. Rectal administration of biologics offers potentially reduced therapy costs, as well as safer and more effective local delivery to inflammation sites. Hydrogels are potentially useful carriers of biologics for improved delivery to the inflamed intestinal mucosa. Here, we prepared a hydrogel system based on ascorbyl palmitate (AP) and incorporated a model macromolecular drug (fluorescently-labelled dextran) into the system. Characterization of gel properties included rheology, drug loading and release, cytotoxicity, and drug delivery in an in vitro intestinal model. We report that this hydrogel can be formed under a moderate environment that is amenable to incorporation of some biologics. The system showed a shear-thinning behavior. AP hydrogel released approximately 60% of the drug within 5 h and showed reasonable a cytotoxicity profile. The study therefore provides evidence that AP hydrogel has potential for local delivery of macromolecules to the intestinal mucosa in IBD.

## 1. Introduction

Crohn’s Disease (CD) and Ulcerative Colitis (UC) are two main forms of inflammatory bowel disease (IBD) affecting an estimated 2.5–3 million people in Europe [1] and with an increasing prevalence in newly industrialized countries. These are chronic, disabling and progressive diseases, impacting every aspect of the affected individual’s life and accounting for substantial costs to the healthcare system and society [2]. Current non-biologic therapy provides symptomatic improvement but fails to influence the underlying inflammation and disease course. Biologics have changed the management of IBD, but parenteral (injection-mediated) administration of these agents is associated with serious limitations. Firstly, systemic toxicity remains a concern [3]. Secondly, patients treated with systemically-administered biologics may experience a loss of sustained response to therapy over time, resulting in symptom flares [4]. The mechanisms behind this are not completely clear, but the gradual development of antibodies to the systemically-administered agent is thought to play a key role. Thirdly, injections often require administration by healthcare professions, adding to healthcare costs.

Key to the development of safer and more effective therapies for IBD is local drug delivery to the sites of inflammation, achieving high drug concentrations locally at the site of inflammation, while minimizing drug availability in the systemic circulation. Enemas offer a means to achieve local drug delivery to the inflamed colon and are routinely used clinically in mild-to-moderate colitis. However, patients with active distal colitis are often unable to retain liquid enemas for extended periods of time [5]. While foam-based enemas may be easier to retain, they are unable to reach tissue areas that are often accessible with a liquid enema [6].

Considering the drawbacks of current enema formulations, there have been a number of advances on improving formulation properties. Recently, a mucoadhesive hydrogel based on catechol modified-chitosan was shown to improve the efficacy of rectal sulfasalazine (small drug molecule) administration in a mouse model of UC [7]. Compared to oral treatment, rectal hydrogel-mediated sulfasalazine delivery was more therapeutic and induced a lower plasma concentration of a potentially toxic drug by-product. In another recent study, a thermo-sensitive system based on a non-ionic surfactant copolymer consisting of hydrophilic polyethylene glycol and hydrophobic polypropylene glycol blocks was developed for rectal budesonide delivery. This system, which is liquid at room temperature but turns into a viscous gel upon reaching body temperature, demonstrated greater colonic retention [8]. In yet another study, an ‘inflammation-targeting’ hydrogel based on ascorbyl palmitate (AP), an amphiphile that is generally recognized as safe (GRAS) by the United States (U.S.) Food and Drug Administration, was developed by Zhang et al [9]. This system delivered the anti-inflammatory corticosteroid dexamethasone in a targeted manner to the site of inflammation owing to its negative charge interacting with the positively charged proteins accumulated selectively to the sites of inflammation in the colonic mucosa [10,11].

While it is clear that current research activity is attempting to improve enema formulations, this activity is mainly focused on the local delivery of small drug molecules. Effective local delivery of biologics, via rectal administration, would potentially offer safer and more clinically-relevant drug delivery to the sites of inflamed tissue, as well as reduce the therapy costs arising from injections. Although the major challenge associated with rectal administration of biologics—that of poor absorption of macromolecules across the intestinal mucosa [12,13]—remains, significant progress has been made in this field, for example, via the use of permeation enhancers [14,15,16] and nanotechnology [12,17,18].

Hydrogels are potentially useful delivery vessels for rectal administration of biologics as they can be formulated from safe and inexpensive materials. We report here that the AP hydrogel can be formed under a moderate environment that is amenable to incorporation of some biologics, such as antibodies, in the system and displays behaviors suitable for rectal delivery of biologics. Specifically, the hydrogel displayed shear-thinning behavior, sustained release of a model macromolecular drug, and acceptable toxicity to intestinal cells. The present study therefore provides evidence that the AP-based hydrogels have potential for rectal delivery of biologics in IBD.

## 2. Materials and Methods

Fluorescein isothiocyanate (FITC)-dextran of average molecular weight 3000–5000 Da (FD4) and dimethyl sulfoxide (DMSO) were purchased from Sigma-Aldrich (Poole, UK). Ascorbyl palmitate (AP) was provided by LKT Labs (St. Paul, MN, USA). Transwell cell culture inserts were obtained from Corning (New York, NY, USA). Hank’s Balanced Salt Solution (HBSS), trypsin, phosphate-buffered saline (PBS), Dulbecco’s Modified Eagle Medium (DMEM), and Triton X-100 were also purchased from Sigma-Aldrich (Poole, UK). AlamarBlue^®^ cell toxicity agent was obtained from Thermo Fisher Scientific (Waltham, MA, USA).

### 2.1. Preparation of Hydrogels

AP hydrogel was prepared at 5% and 6% *w*/*v* using DMSO and water (1:4). AP was dissolved in DMSO in a glass vial by heating to 40 °C or 50 °C with stirring. Subsequently, water warmed to the same temperature was added dropwise to the AP solution under stirring. The vial was subsequently cooled to room temperature. It must be noted that AP hydrogels were also prepared at 7% and 8% *w*/*v*, but these systems displayed very high viscosities, leading to difficulties in mixing in the DMSO and water phase.

To prepare FD4-loaded AP hydrogel, FD4 was dissolved in water and this solution was added to the AP:DMSO solution dropwise.

### 2.2. Rheological Characterization

The rheological assessment of 5% and 6% *w*/*v* AP hydrogels was performed through a 35 mm diameter and 4° cone-and-plate rheometer (Physica MCR 51, Anton Paar, Austria). The samples were placed into the gap between the cone and plate to control the temperature. Three independent experiments were performed for each sample using separately prepared AP hydrogel batches. The rheometer was set to 20 °C to measure the change in viscosity as a function of shear rate. Measurements were conducted around room temperature to simulate conditions of local application (rectal) of the system. The range of shear rate was from approximately 1 s^–1^ to 100 s^−1^. The rheological profile was analyzed by plotting the viscosity-shear rate profile.

### 2.3. Drug Release from Hydrogels

For drug release studies, cell culture inserts were used as filters on which the hydrogels were applied at room temperature. First, 50 mg of 5% *w*/*v* FD4-loaded hydrogel was applied on the insert membrane (donor side), followed by the addition of 0.5 mL HBSS. Then, 1.5 mL HBSS was added to the acceptor compartment. At 0, 0.5, 1, 1.5, 2, 3, 4, 5, 6, and 7 h, 100 μL of the solution from the acceptor compartment was sampled and transferred to a black 96-well plate. The sampled solution was replaced with fresh HBSS. FD4 was quantified by fluorescence at 485/20 nm excitation wavelength and 540/30 nm emission wavelength using a fluorescence multiwell plate reader (Series 4000, CytoFluor). The same procedure was repeated for the 6% *w*/*v* FD4-loaded hydrogel. Three repeats were performed for each sample. FD4 release is expressed as percentage cumulative release over time.

### 2.4. Cytotoxicity Assay

Caco-2 cells were seeded at 5 × 10^4^ cells per well in 96-well plates. Cells were grown for 72 h in DMEM. Then, 5% *w*/*v* AP hydrogel was suspended in HBSS and added to the cells at different concentrations. HBSS was used as the negative control and Triton X-100 (0.2% *v*/*v*) as the positive control (inducing cell death). Cells were incubated with the samples for two h. Following this, cell viability was determined via the AlamarBlue reagent (diluted 1:10 in HBSS) and measurement of fluorescence at 560/20 nm excitation wavelength and 590/30 nm emission wavelength according to the supplier’s instructions. Cell viability is reported relative to HBSS and Triton X-100.

### 2.5. Permeability Study

Caco-2 cells were seeded at 1 × 10^5^ cells/well on Transwell inserts and cultured for 21 days. Prior to the permeability study, culture medium was replaced with HBSS; cells were equilibrated in this for around 40 minutes. FD4-loaded hydrogel (5% *w*/*v* AP) was diluted to 250 mg/mL (gel content) in HBSS and applied to the cells at this concentration. Basolateral solution was sampled (100 μL volumes) periodically for two h and FD4 was quantified by fluorescence. A parallel experiment was conducted with free FD4 application to Caco-2 monolayers at equivalent concentration. Experiments were performed in triplicates.

## 3. Results

### 3.1. Hydrogel Preparation

**Figure 1:**
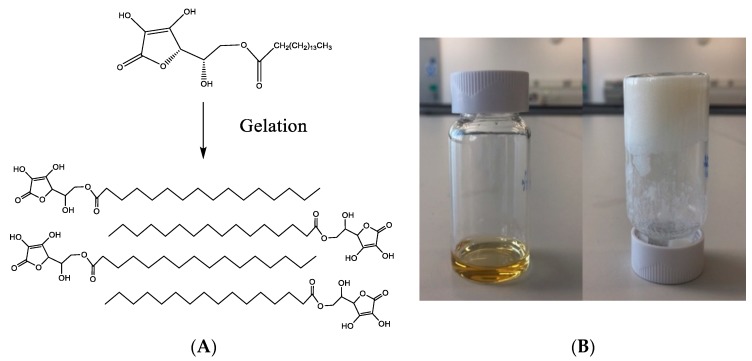
Formation of ascorbyl palmitate (AP) hydrogel. (**A**) Molecular structure of AP and the assembled hydrogel, highlighting the central alignment of the hydrophobic portion and the hydrophilic heads on the outside of the bilayer (adapted from Zhang et al. [9]). (**B**) AP dissolved in dimethyl sulfoxide (left) and addition of water to this solution resulting in hydrogel (right).

The gelation conditions were investigated at different temperatures and concentrations of AP. When the mixture was prepared at 40 °C and cooled to room temperature, no hydrogel was formed (data not shown). Increasing the temperature to 50 °C resulted in successful gelation, as shown in Figure 1. Therefore, 5% and 6% *w*/*v* AP hydrogels were subsequently prepared at 50 °C.

### 3.2. Rheological Characterization

**Figure 2:**
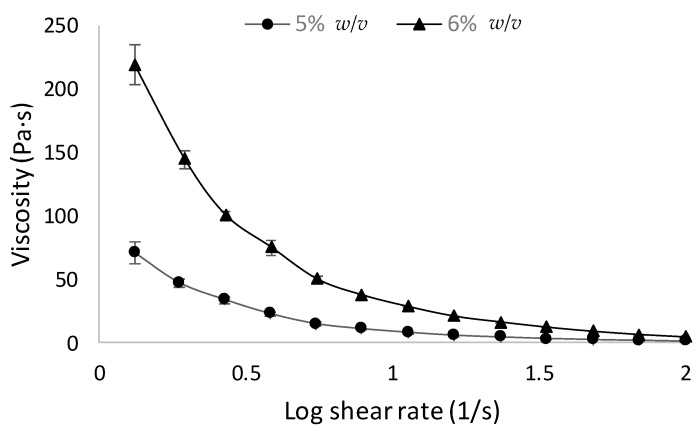
Rheological properties of 5% and 6% *w*/*v* ascorbyl palmitate hydrogels at 20 °C. Three independent studies per sample were performed. Data shown as means ± SD.

The rheological profiles of the AP hydrogels are shown in Figure 2. Viscosity was dependent on the concentration of AP, with the 6% *w*/*v* hydrogel being more viscous than the 5% *w*/*v* counterpart (Figure 2). Viscosity decreased with an increase in shear rate for both AP concentrations. Data therefore demonstrated that the AP hydrogel displays a shear-thinning behavior.

### 3.3. Drug Release from Hydrogels

**Figure 3:**
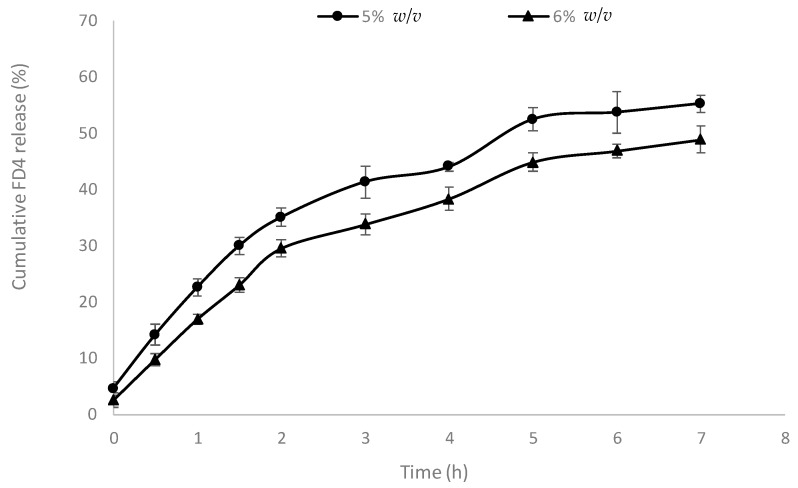
Drug release profile of ascorbyl palmitate hydrogel. Drug release was tested in Hank’s Balanced Salt Solution at room temperature. Data shown as mean ± SD (*n* = 3).

Studies evaluating the release profile of FD4 (used as a model macromolecular drug) from the AP hydrogel were conducted in HBSS as a physiological buffer. The hydrogel was placed on a filter (Transwell inserts) to support the hydrogel, and the released FD4 (capable of passing through the filter pores) was sampled from the acceptor compartment. Figure 3 shows a sustained release of FD4 from the hydrogels. FD4 release from the 5% *w*/*v* AP hydrogel reached 52.42% within five h of the study, followed by a plateau for the subsequent measurement period. FD4 release peaked at approximately 55%. FD4 release from the 6% *w*/*v* AP hydrogel peaked at approximately 49% in seven h. Considering the higher drug release from the 5% hydrogel, this system was selected for subsequent cell culture experiments.

### 3.4. Cell Toxicity of Ascorbyl Palmitate Hydrogel

**Figure 4:**
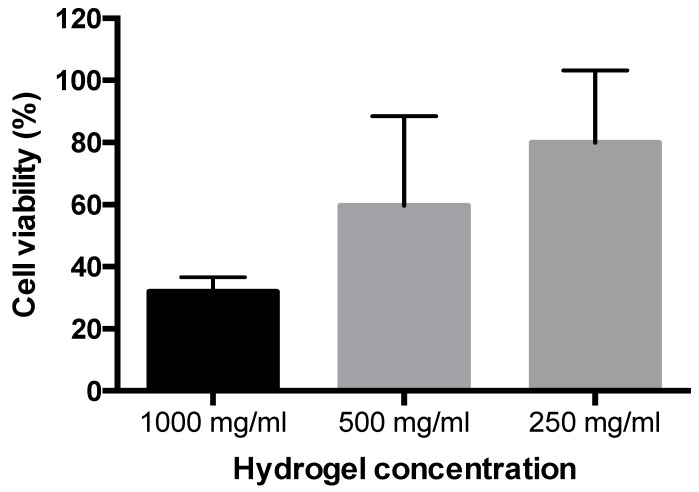
Effect of ascorbyl palmitate (AP) hydrogel on Caco-2 cell viability. AP hydrogel was applied to cells for two h at different concentrations in Hank’s Balanced Salt Solution. Viability was measured via the AlamarBlue reagent. Data shown as mean ± SD (*n* = 3).

Intestinal Caco-2 cells were used to evaluate the toxicity of the 5% *w*/*v* AP hydrogel. Figure 4 indicates that cytotoxicity was concentration-dependent, although this was just outside the statistical significance (*p* = 0.065). Cell viability was as high as 80% following the application of a high hydrogel concentration of 250 mg/mL (i.e. 12.5 mg/mL AP).

### 3.5. Permeability Study

**Figure 5:**
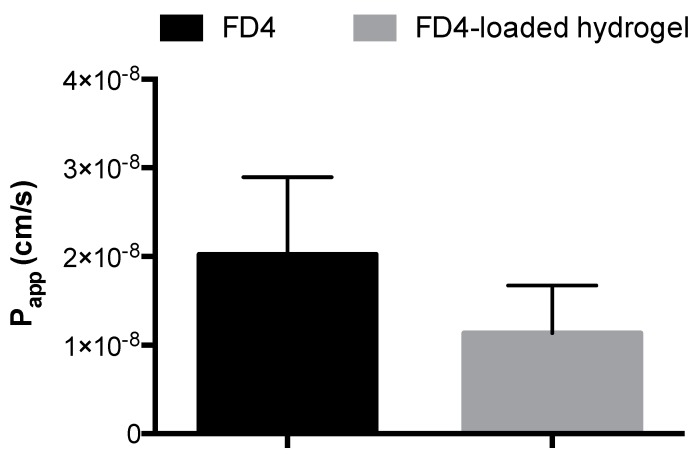
Permeability of fluorescein isothiocyanate (FITC)-labelled dextran of ~4 kDa (FD4) when applied to Caco-2 monolayers in solution (‘FD4’) or loaded in hydrogel (‘FD4-loaded hydrogel’). Hydrogel formulated from 5% *w*/*v* ascorbyl palmitate. Data shown as the mean ± SD (*n* = 3, *p* > 0.05).

The permeability of FD4 loaded on 5% *w*/*v* AP hydrogel across Caco-2 cell monolayers was tested to assess the potential of the hydrogel for intestinal delivery of macromolecules. FD4-loaded hydrogel was compared with free FD4 (applied at equivalent concentration); the difference in permeability between these conditions was not statistically significant (Figure 5).

## 4. Discussion

Local delivery of biologics in IBD presents an opportunity to achieve therapeutic delivery at high doses to inflammation sites, whilst significantly reducing the systemic exposure and related negative consequences (systemic toxicity and loss of response). Topical therapies delivered via the rectal route offer one way of achieving such local drug delivery. Enemas are routinely used clinically in mild-to-moderate colitis and can successfully treat acute flares, as well as maintain remission for many patients. It is thought that over half of patients with UC and a smaller fraction with Crohn’s colitis may benefit from topical therapy alone, given that their disease is limited to the distal colon/rectum [8].

Liquid enemas are often not tolerated by patients with active distal colitis due in part to urgency and the associated inability to retain them [5]. While foams may be easier to retain, they are unable to reach proximal areas of the left colon [6]. These issues contribute to low patient adherence [8]. Given the potential of topical therapy in IBD, it is somewhat surprising that, given the progress in fabrication of novel drug delivery systems for other routes of administration in IBD [10], significant breakthroughs in new and improved systems for rectal administration are, to the best of our knowledge, not apparent. Some exceptions include recently-reported systems based on mucoadhesive or thermo-responsive hydrogels [7,8,19]. However, these have been designed and shown potential for rectal delivery of small drug molecules rather than macromolecules.

The rectal route of drug administration is often overlooked due to concerns with patient acceptability [20] and adherence [21]. However, the potential advantages of locally-delivered drug therapy in IBD (improved safety and efficacy), coupled with infrequent dosing requirements for some biotherapeutics (e.g. siRNA) [22], warrant focus in this area.

Hydrogels are potentially useful vehicles for incorporation and delivery of biological therapeutics to the inflamed mucosal surfaces. Previous studies have investigated a plethora of hydrogels for intestinal delivery of therapeutic proteins such as interferon beta [23] and calcitonin [23,24]. However, exploration of hydrogels as vehicles for delivery of biologics has focused on the oral route. We, on the other hand, investigated a recently-reported hydrogel for its potential utility as a drug delivery system for rectal administration of biological therapeutics in IBD.

The AP hydrogel investigated here was recently reported as a novel, inflammation-targeting hydrogel for application, via rectal administration, in IBD [9]. AP consists of a partially charged, hydrophilic head (ascorbic acid) and a hydrophobic tail (palmitic acid), linked by an ester bond (Figure 1A). In a DMSO/water mixture, AP is organized into extended micellar structures and interdigitated bilayers with a hydrophobic core and hydrophilic outer layer, forming a fibrous structure. Following the dissolution of AP with heating, gelation takes place when the mixture cools to room temperature [9]. It is claimed that this system has several potential advantages, including composition from a nontoxic, ‘generally recognized as safe’ (GRAS) compound with simple and scalable formulation (gelation process), high drug-loading capacity, and long-term stability, which enables sustained drug release [9]. The latter was attributed to the microstructure of the gel fibers offering protection from spontaneous hydrolysis by water, which cannot penetrate efficiently into the hydrophobic fiber core.

To investigate the potential of AP hydrogel for delivery of macromolecules we modified the fabrication conditions by reducing the temperature. Gelation was clearly apparent at 50 °C (but not at 40 °C). These conditions may be amenable to formulation of AP hydrogel loaded with therapeutic antibodies, which, compared with other proteins, are more resistant to thermal stress [25]. The AP hydrogel also displayed a shear-thinning behavior (Figure 2), which is a useful characteristic for rectal delivery [26], promoting colonic distribution upon administration.

The release of the FD4 model macromolecular drug from the hydrogel peaked at approximately 49% and 55% of the loaded content for 6% and 5% *w*/*v* hydrogel, respectively, within seven h (Figure 3). It is apparent that the AP hydrogels exhibited a small degree of burst release (evident by 30% and 35% release of FD4 in the first two h for the 6% and 5% AP hydrogel, respectively). However, it is presently unclear how this release behavior is influenced by the properties of the macromolecular cargo, including its molecular weight. While burst release is undesirable for systemic drug delivery (due to potential toxicity), for local mucosal delivery of macromolecules that are poorly absorbed into the systemic circulation, it is envisaged that the presence of some burst release is not problematic. The overall prolonged drug release exhibited by the system is advantageous for sustained presentation of the drug to the mucosal surface, particularly considering the likely prolonged residence of the hydrogel at mucosal sites of inflammation arising from charge interaction [9]. The 5% *w*/*v* hydrogel with lower viscosity displayed a higher drug release and was selected for cell culture experiments.

Cell toxicity studies intentionally utilized high concentrations of the hydrogel. At an applied hydrogel concentration of as high as 250 mg/mL, corresponding to 12.5 mg/mL AP, intestinal epithelial cell viability was only modestly reduced (by approximately 20%, Figure 4). Due to the novelty of the system, we can only compare our observations with those of Zhang et al. [9], where this gel was added to Caco-2 cells at a maximal concentration of 16 μg/mL of in culture medium, and no toxicity was apparent. Future studies should however study the potential toxicity of the AP hydrogel more comprehensively considering the DMSO content of the system.

Final evaluation of the AP hydrogel addressed the release of FD4 and its permeability across intestinal Caco-2 cell monolayers as conditions modelling macromolecule release and intestinal delivery. The data indicated that FD4 was released from the system and permeated the Caco-2 intestinal epithelial monolayers to a similar extent as free FD4 (Figure 5). It is noted that, following rectal delivery, biotherapeutic macromolecules are likely to demonstrate poor epithelial penetration, but this issue can be addressed by incorporation of permeation enhancers into the system.

## 5. Conclusions

In conclusion, this study demonstrated that the AP hydrogel is a system with highly desirable properties for potential use as a vehicle for rectal delivery of biologics, and future studies should continue to comprehensively evaluate its potential for this application.
